# A Human ECG Identification System Based on Ensemble Empirical Mode Decomposition

**DOI:** 10.3390/s130506832

**Published:** 2013-05-22

**Authors:** Zhidong Zhao, Lei Yang, Diandian Chen, Yi Luo

**Affiliations:** 1 College of Electronics and Information, Hangzhou Dianzi University, Hangzhou 310018, China; 2 College of Communication Engineering, Hangzhou Dianzi University, Hangzhou 310018, China; E-Mails: yangleihd09@163.com (L.Y.); hainingcdd@163.com (D.C.); luoyi@hdu.edu.cn (Y.L.)

**Keywords:** biometrics, ECG Identification System, ensemble empirical mode decomposition, k-nearest neighbors

## Abstract

In this paper, a human electrocardiogram (ECG) identification system based on ensemble empirical mode decomposition (EEMD) is designed. A robust preprocessing method comprising noise elimination, heartbeat normalization and quality measurement is proposed to eliminate the effects of noise and heart rate variability. The system is independent of the heart rate. The ECG signal is decomposed into a number of intrinsic mode functions (IMFs) and Welch spectral analysis is used to extract the significant heartbeat signal features. Principal component analysis is used reduce the dimensionality of the feature space, and the K-nearest neighbors (K-NN) method is applied as the classifier tool. The proposed human ECG identification system was tested on standard MIT-BIH ECG databases: the ST change database, the long-term ST database, and the PTB database. The system achieved an identification accuracy of 95% for 90 subjects, demonstrating the effectiveness of the proposed method in terms of accuracy and robustness.

## 1. Introduction

With the development of society and technology, traditional human identification technologies like keys, ID cards, and passwords are no longer adequate to satisfy modern security demands. The advent of biometric identification technology (BIT) may effectively solve this problem. BIT is a technology that uses physiological or behavioral characteristics extracted from human subjects to automatically identify the individual subject by using a computer-based algorithm [1]. In the past few decades, a number of biometric identification technologies have been investigated, based on physiological characteristics such as the face, iris, retina, and fingerprints, and behavioral characteristics such as speech, signature, or gait. Although these biometrics offer unique advantages such as portability, reliability, and security, they also have unique defects, including the ease with which they can be circumvented, for instance by using a prosthetic finger or iris, or a facial photo.

Because ECGs reflect the cardiac electrical activity and contain substantial important information about the human body, they have been used as a tool for clinical diagnosis since the early 20th century. Recently, researchers have evaluated ECGs as a new biometric characteristic for human identification [2–13]. The validity of ECG as a biometric identification tool is supported by its permanence, universality, and uniqueness. Every living person produces ECG signals, which can be observed and recorded throughout their lifetime. Individual differences in heart physiology and geometry yield produce unique features in ECG signals. The ECG signal has several advantages compared to other biometric characteristics: the heart rate, and therefore the ECG waveform, is controlled by the autonomic nervous system and is affected by other sympathetic and parasympathetic factors, making it difficult to mimic. Because ECG is a feature generated inside the body, environmental factors cannot significantly impact the waveform. In addition, the primary structure of the signal is stable over a long period of time. The ECG signal is easily collected by placing the two thumbs onto electrode nodes and this inherently indicates the animate nature of the subject.

Identification systems based on ECGs also have the advantages of requiring minimal space and offering rapid results as opposed to other biometrics. Based on the number of extracted multiple-fiducial points, previous work can be divided into three categories: fiducial-based, single-fiducial-based, and non-fiducial-based approaches. Table 1 summarizes these approaches.

### 1.1. Multiple-Fiducial-Based Approach

Multiple-fiducial-based approaches extract features from selected points on the ECG waveform, such as the duration and amplitude of the P and T waves and the QRS complex, and then apply designated classifiers for identification.

Biel *et al.* extracted multiple-fiducial features from each standard 12-lead ECG signal to obtain 30 characteristics including the width, duration, and amplitude of the P and T waves and the QRS complex [2]. Principal component analysis (PCA) was used to reduce the dimensions of the feature coefficients. Finally, the soft independent modeling of class analogy model was used for classification, with a final identification rate of 100% over 20 healthy subjects. Later, Biel proposed another method based on a single-lead ECG signals.

Shen *et al.* extracted seven temporal and amplitude features from the QRST wave [4], and then used a template matching method to calculate the correlation coefficients of two QRS complexes to classify the subjects. The possible candidates resulting from the template matching were put into a decision-based neural network (DBNN), with a final recognition rate of 100% over 20 subjects. They later evaluated the system using a larger database containing 168 subjects and 17 temporal and amplitude features, with a resulting system accuracy of 95% [3].

Wang *et al.* combined analytic and appearance features extracted from ECG fiducials to achieve a high identification rate [5]. PCA and linear discriminant analysis (LDA) were used to filter the redundancy features. In a database consisting of 29 participants, the recognition accuracy was 100% and the heartbeat recognition rate was 92.4%.

Li Wang employed a method based on wavelet noise reduction to eliminate the effects of noise [6]. The difference threshold method was used to extract eight duration and amplitude features from the P and T waves and QRS complex. The feature coefficients were fed into the back propagation (BP) and radial basis function (RBF) neural networks, for an identification rate of 100% in a database of 10 individuals.

The disadvantage of multiple-fiducial-based approaches is that their identification performance is affected by the accuracy of fiducial point detection. Furthermore, there is no universal standard defining the boundaries of the ECG wave features. Fiducial points detected by ECG devices are approximate locations and do not satisfy biometric system requirements because even slight variation in the locations will result in misclassification. The P and T waves are sometimes too small to detect, preventing exact extraction of fiducial features such as the duration and width of the P and T waves.

### 1.2. Single-Fiducial-Based Approaches

Because the R point is easy to identify in the ECG signal, single-fiducial based approaches extract the heartbeat cycle signal based on the R point location and use the transformed waveform or coefficients as the features.

Chiu *et al.* extracted a fixed-length heartbeat signal based on the position of the R point [7]. Wavelet transformation was used to decompose four continuous heartbeat signals and obtain the feature coefficients. The identification rate was 100% over 30 healthy individuals and 81% over in a database of 30 healthy subjects and eight unhealthy subjects.

Chan *et al.* extracted a fixed-length heartbeat signal based on the position of the R point and using its wavelet coefficients as the features [8]. A threshold was then selected according to the correlation analysis. The system was evaluated using a database of 30 individuals with an accuracy of 82.5% and 87.5%, respectively, when combined the two features, and the identification rate is 95%.

The identification performance of single-fiducial-based approaches is not affected by the accuracy of fiducial point detection, but these methods extract feature coefficients based on a fixed-length heartbeat signal and do not consider the effects of heart rate variability (HRV). In general, the normal heartbeat range is 60–100 bpm, but it changes with heart rate.

### 1.3. Non-Fiducial-Based Approaches

To eliminate the need for fiducial point localization in the ECG signal, Plataniotis *et al.* used an autocorrelation (AC) of the segmented ECG signal followed by discrete cosine transform (DCT) or LDA [9,10,13], also called the AC/DCT or AC/LDA methods, with identification accuracies of 92.8% and 100%, respectively. Although the AC/DCT and AC/LDA methods achieved high recognition rates, these methods also have disadvantages. These approaches do not use ECG data effectively and require ECG data of a minimum length to calculate the AC coefficients; however, they do capitalize on the information in the heartbeat signal.

This paper describes a new feature extraction method based on ensemble empirical mode decomposition (EEMD) for automated analysis of single-lead ECGs in biometric identification applications. The proposed system applies a robust preprocessing algorithm to the raw ECG signal to compensate for noise and heart rate variability (HRV), followed by EEMD and Welch spectral analysis to extract the important temporal and spectral features from one heartbeat signal. Furthermore, the K-nearest neighbors (K-NN) method was used for classification after application of the PCA dimensionality reduction technique to increase the processing speed.

## 2. Electrocardiogram Data and Noise

### 2.1. Electrocardiogram

**Figure 1.**
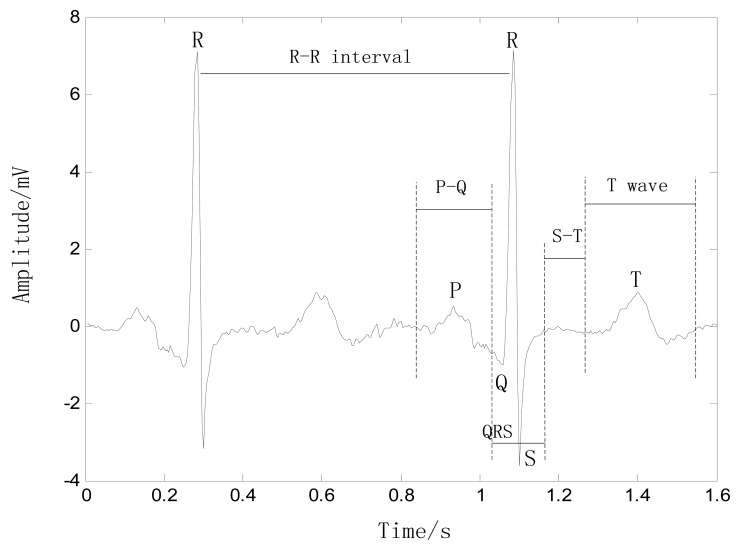
Classical wave profile of an ECG signal.

An ECG trace describes the electrical activity of the heart as recorded using electrodes placed on the body surface. The variation in voltage is due to the action potentials of cardiac cells. The sinoatrial (SA) node is the pacemaker of the heart, and hence, it is responsible for regulation of the heart rate. The electrical activity is initiated when the SA node depolarizes, and the electrical impulse travels rhythmically along the conduction pathway, stimulating sequential contraction and relaxation of the heart muscle. The final destination is the atrioventricular (AV) node, which is responsible for regulating the conduction rate to properly pump blood from the atria into the ventricles [14,15]. The resulting heartbeat measured is by the ECG as a series of waves with a particular morphology, rhythm, and rate, as shown in Figure 1, which come from an ECG data on MIT-BIH database [16].

P wave: This wave reflects the depolarization of the right and left atria, and has a smooth morphology and small amplitude because of the limited atrial muscle mass. The spectrum of the P wave is considered to be about 10–15 Hz. When the heart rate changes, the magnitude and duration of the P wave also changes slightly.

QRS complex: This complex has the largest amplitude of the ECG waveform and is the most important part of the ECG signal. Because of its steep slope, the QRS complex has a high-frequency spectrum concentrated in the range 10–50 Hz. The QRS complex is considered to be fairly constant and is not altered by changes in heart rate, as it reflects the time between the depolarization of the right and left ventricles.

S-T interval: This interval begins at the end of the S wave and curves into the T wave, representing the interval during which the ventricles remain in an active, depolarized state. When the heart rate changes, the S-T interval is altered significantly.

T wave: This wave reflects the depolarization of the ventricles, and its position relies strongly on the heart rate, appearing closer to the QRS complex at rapid rhythms. The T wave shifts significantly with changes in the heart rate, e.g., when the heart rate increases, the magnitude of the T wave increases and the duration decreases, with an almost linear correlation to heart rate.

### 2.2. ECG Noise

During the ECG recording, the signal may be corrupted by low- and high-frequency noise that alters the waveform of the ECG trace from its original structure. To eliminate this noise, the most common types of noise and artifacts must be considered [14,15].

*Quality measurement:* Extraneous noise in the ECG trace may be caused by a variety of noise sources including perspiration, respiration, body movements, and poor electrode contact. The magnitude of this noise may exceed the amplitude of the QRS complex by several times, but its spectral content is usually confined to an interval below 1 Hz.

*Electrode motion artifacts:* These are manifested as large-amplitude waveforms primarily caused by stretching that alters the impedance of the skin around the electrode. They are more difficult to combat because their spectral content ranges from 1 to 10 Hz and overlaps considerably with the PQRST complex.

*Power line interference (50/60 Hz):* This is high-frequency noise caused by interference from nearby devices, resulting from improper grounding of the ECG equipment.

*Electromyography noise (EMG noise):* EMG noise is caused by the electrical activity of skeletal muscles during periods of contraction or owing to a sudden body movement. Although, the frequency component of EMG overlaps considerably with that of the QRS complex, it also extends into higher frequencies. As a result, the processing of the ECG trace to remove these noises effects naturally results in some distortion of the signal.

*Respiratory activity:* Beat-to-beat variation in the morphology and heart rate occurs during the respiratory cycle either as a result of changes in the dominant vector direction of the electrical wave propagation due to changes in heart position or as a result of changes in lung conductivity.

## 3. Theoretical Framework

### 3.1. EMD Algorithm

Based on the assumption that any signal comprises intrinsic mode functions (IMFs) of different scales, the EMD method can decompose a signal into a set of IMF components. An IMF is a function that satisfies the following conditions: (1) the number of extremes and the number of zero-crossings in the data set must either be equal or must differ by no more than one; and (2) at any point, the mean value of the envelope defined by the local maxima and the envelope defined by the local minima is zero. The EMD processing of a signal *x(t)* can be described as follows [17]:

(1) Initialize *r_0_* = *x(t)*, where *i* = 1;
(2) Extract the *i*^th^ IMF;
  (a) Initialize *h_i_(k−1)* = *r_i_*, where *k* = 1;
  (b) Extract the local maxima and minima of *h_i_(k−1)*;
  (c) Interpolate the local maxima and minima using a cubic spline function to construct the upper and lower envelopes of *h_i_(k−1)*;
  (d) Calculate the mean *m_i_(k−1)* of the upper and lower envelopes of *h_i_(k−1)*;
  (e) Let *h_ik_* = *h_i_(k−1) − m_i_(k−1)*;
  (f) If *h_ik_* is an IMF, then set *c_i_* = IMF*i* = *h_ik_* or else go to step (b) where *k* = *k* + 1;
(3) Define *r_i_*_+1_ = *r_i_−* IMF*i*; and
(4) If *r_i_*_+1_ has at least two extrema, then go to Step (2), otherwise the decomposition process is stopped and *r_i_*_+1_ is the residue of the signal.

The result is the residue *r_l_*_+_*_1_* and the collection of l IMFs *c_i_* (i = 1,2,3, …, 1). The summation of all the IMFs and the final residue *r_l_*_+_*_1_* yields:

(1) $$x\left(t\right)=\sum_{i=1}^{l}c_{i}+r_{l+1}$$

Thus, the signal x(t) is decomposed into l IMFs and a residue *r_l_*_+_*_1_*, which represents the mean trend of the signal x(t). The IMFs *c_1_, c_2_*,…, *c_l_* represent different frequency components from high frequency to low frequency, whereas *r_l_*_+_*_1_* indicates the general tendency of the signal x(t).

### 3.2. EEMD Algorithm

One shortcoming of the EMD method is the appearance of mode mixing. Mode mixing is defined as a single IMF including oscillations of dramatically disparate scales, or a component of a similar scale residing in different IMFs. It is a result of signal intermittency. As discussed by Huang *et al.* the intermittence could not only cause serious aliasing in the time–frequency distribution, but also make physical meaning of individual IMF unclear. When the mode mixing problem occurs, an IMF can cease to have physical meaning by itself, suggesting falsely that there may be different physical processes represented in a mode. To overcome the problem of mode mixing in EMD, a new noise-assisted method called EEMD is proposed as a solution to the mode mixing problem of the EMD method. EEMD defines the true IMF components as the mean of an ensemble of trials, each comprising the signal with an added white noise component of finite amplitude. The ensemble method can clearly separate the natural scale of signals without the need for subjective *a priori* criteria. Recent studies on the statistical properties of white noise have suggested that EMD is an effective self-adaptive dyadic filter bank when applied to white noise, which inspired the new method [18–20].

The principle of EEMD is as follows: the added white noise constitutes components of different scales that uniformly inhabit the entire time-frequency space. When a signal is added to the uniformly distributed white noise background, the different scale components of the signal are automatically projected onto proper scales of reference established by the white noise component. Because each of the decompositions contains the signal and the added white noise, each individual trial generates very noisy results. Because the noise in each trial is different, it can be almost entirely removed by calculating the ensemble mean of all trials. The ensemble mean is treated as the true answer because only the signal is preserved as the number of trials added to the ensemble increases. The essential principle of the proposed method is based on the following observations [20]:

(1) A uniformly applied white noise background is cancelled out in a time-frequency ensemble mean; therefore, only the signal remains in the final noise-added ensemble mean.
(2) White noise of finite amplitude necessarily compels the ensemble to discover all possible solutions. The white noise causes the different scale signals to reside in the corresponding IMFs, controlled by dyadic filter banks, and renders the results of the ensemble mean more meaningful.
(3) The EMD result with true physical meaning is not one without noise; however, it is the ensemble mean of many trials using noise-added signals.

Based on the aforementioned observations, the EEMD algorithm can be stated as follows:

(1) Execute the *m*^th^ trial for the signal with added white noise.
  (a) Add the white noise series with the given amplitude to the investigated signal, *i.e.*
    (2) $${\text{x}}_{\text{m}}\left(\text{t}\right)=\text{x}\left(\text{t}\right){\text{n}}_{\text{m}}\left(\text{t}\right)$$
    where n_m_(t) represents the *m*^th^ added white noise and x_m_(t) indicates the noise-added signal of the *m*^th^ trial.
  (b) Decompose the noise-added signal x_m_(t) into *l* IMFs *c_im_(i* = *1, 2*,…, *l, m* = *1, 2*,…, *M)* using the EMD method, where c_im_ indicates the *i*^th^ IMF of the *m*^th^ trial; *l* is the number of IMFs; and *M* is the number of the ensemble members.
  (c) If *m* < *M*, then let *m* = *m* + *1* and repeat the steps (a) and (b) until *m* = *M*, but using different white noise each time.
(3) Compute the ensemble mean *C̅_i_* of the *M* trials for each IMF to obtain:
  (3) $${\bar{c}}_{i}=\frac{1}{M}\sum_{m=1}^{M}c_{i,m}$$
(4) Report the mean *ĉ_i_*(*i*=1,2, ⋯,*l*) of each of *l* IMFs as the final *i*^th^ IMF.

**Figure 2.**
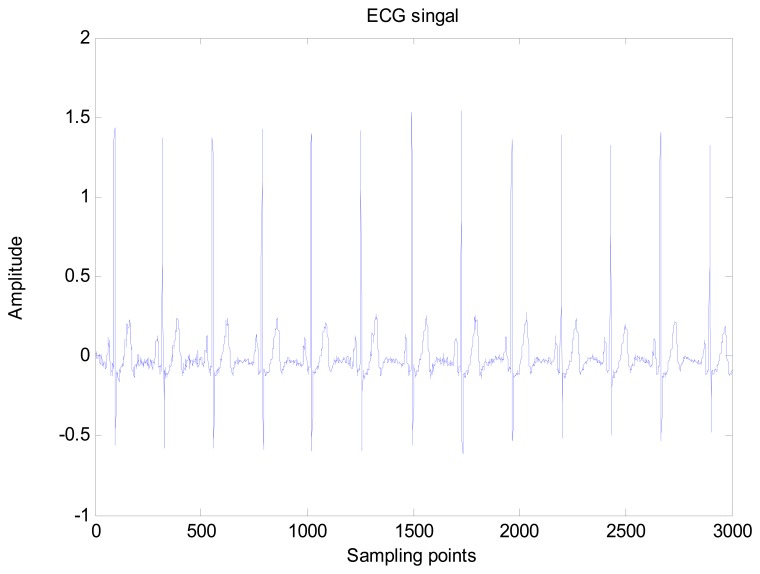
ECG signal.

**Figure 3.**
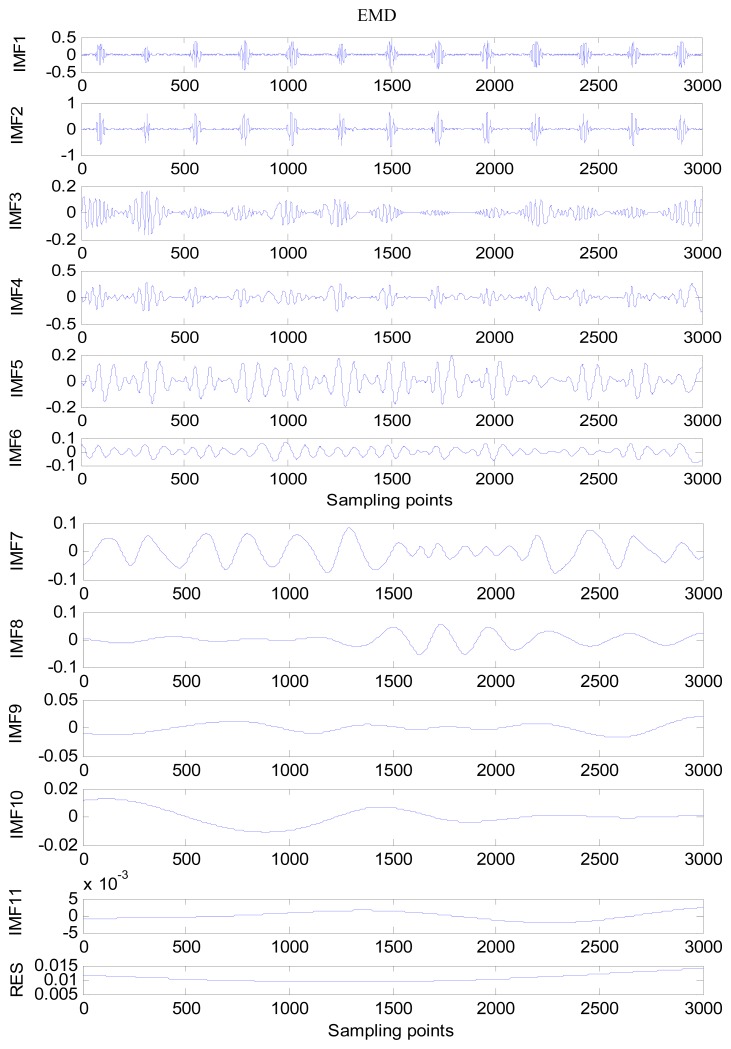
IMFs obtained by EMD. From top to bottom is low level IMF to high level IMF.

An amount of research has been done recently about EMD/EEMD for ECG signal processing [21–26]. To demonstrate the EEMD performance of overcoming the mode mixing problem, an ECG signal is considered in this section. The ECG signal record 16,272 m comes from the MIT-BIH Normal Sinus Rhythm Database. The signal is a length of 3,000 sample points. The signal is decomposed by EMD and EEMD respectively to illustrate the mode mixing problem. The ECG signal is shown in Figure 2, and the decomposed IMFs are shown in Figure 3. Mode mixing phenomenon exists.

**Figure 4.**
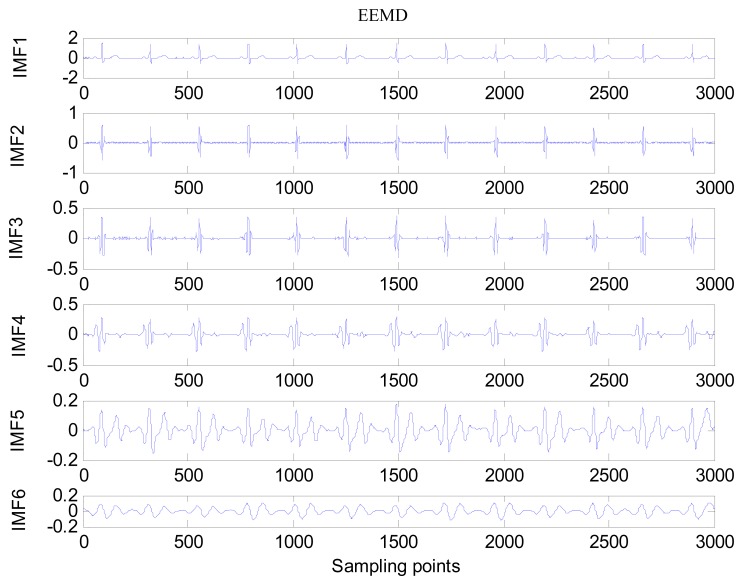

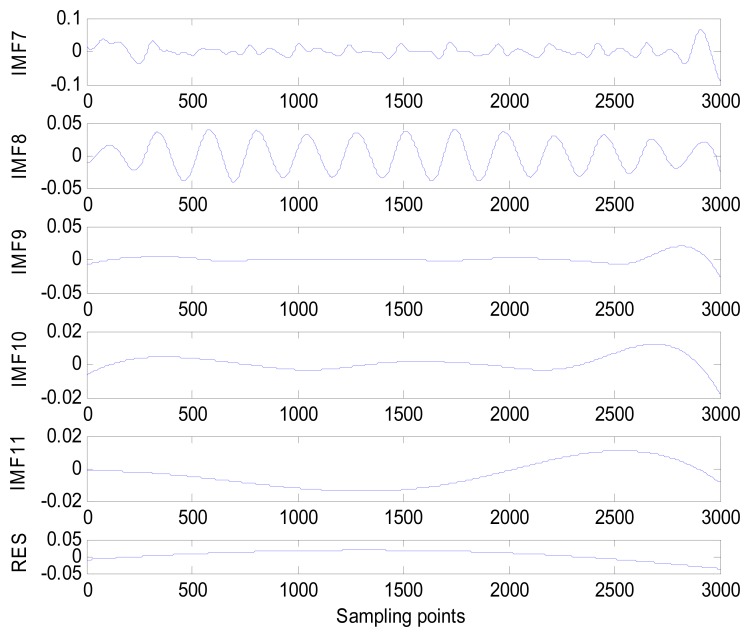
IMFs obtained by EEMD. From top to bottom is low level IMF to high level IMF.

**Figure 5.**
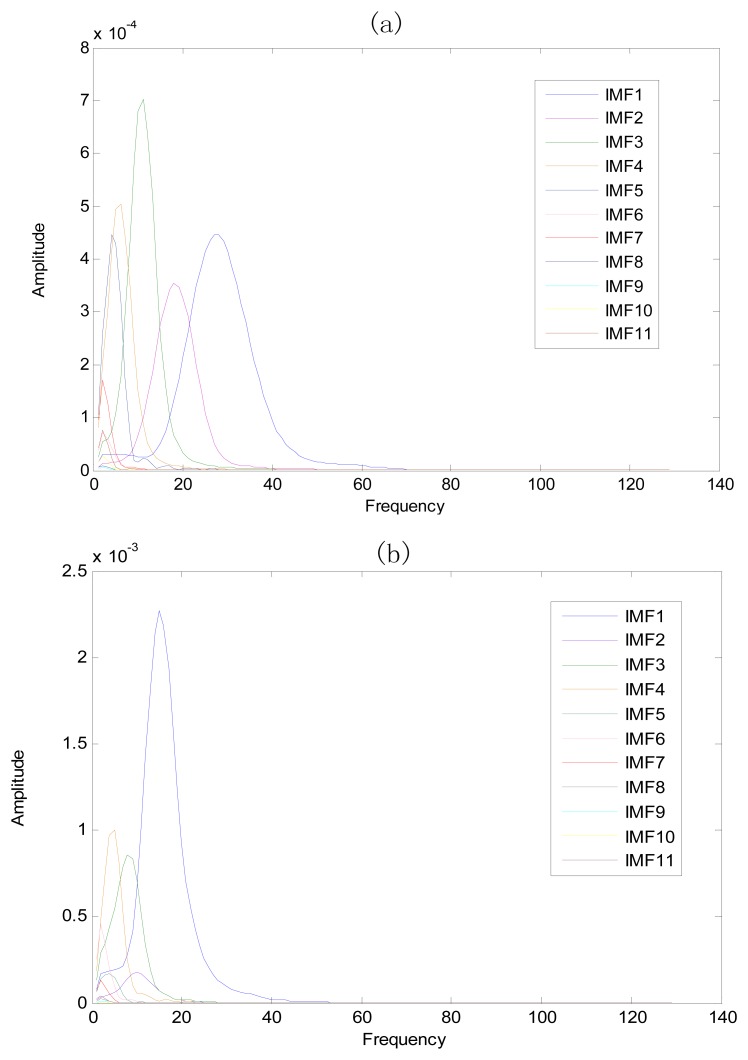
Corresponding IMF spectrum distribution of (**a**) EMD and (**b**) EEMD.

The same ECG signal is decomposed again using EEMD with the ensemble number 30 and the added noise amplitude 0.1 time standard deviation of the signal. The IMFs are shown in Figure 4. The corresponding IMF spectrum distribution of EMD and EEMD are also illustrated in Figure 5. The difference between EMD and EEMD is the mode mixing reduction of EEMD. Comparing the IMF component of the same level, EEMD has more concentrated and band limited components. EEMD method is able to solve the problem of mode mixing and achieve an improved decomposition with physical meaning. The conclusion is identical to reference [27].

### 3.3. EEMD Parameter Settings

The previous section described the EEMD algorithm and its specific operating process. However, before employing EEMD, two parameters must be set: the ensemble number and the amplitude of the white noise.

#### 3.3.1. Ensemble Number

The effect of added white noise should conform to the following statistical rule:

(4) (4) $$e_{n}=\frac{a}{\sqrt{N}}$$

where *N* is the ensemble number, *a* indicates the amplitude of the added white noise, and *e_n_* is the standard deviation of error, which is defined as the discrepancy between the input signal and the corresponding IMFs.

To increase the effectiveness of EEMD, the amplitude of the added white noise must be large enough to generate the change of extrema required for EMD. This is true when the investigated signal has a large gradient. Although the added white noise may result in some errors, its effects can be reduced to a negligible level. Generally, an ensemble number of a few hundred will produce an accurate result, and the remaining noise would cause no more than a fraction of one percent error if the amplitude of the added noise is a fraction of the standard deviation of the investigated signal [20].

#### 3.3.2. Amplitude of Added White Noise

Because it is truly dyadic, EMD decomposition is a noise-friendly method. Within a certain range of noise amplitude, the decomposition results of EEMD have a minimal sensitivity to the noise amplitude. The decomposition results change very little with increasing noise amplitude and ensemble number, provided the added noise has a moderate amplitude and the ensemble number is large enough. However, to reduce the contribution of the added noise to the decomposition results, the ensemble number should increase with the noise amplitude. Therefore, decomposition results using EEMD, different from those of EMD, are unique and robust. The proper amplitude of added noise should be about 0.2 times the standard deviation of the investigated signal, although this is not always possible and there is not a specific principle to guide selection of the noise amplitude. Consequently, it is necessary to try several different noise levels to determine the most appropriate one [20].

### 3.4. Spectral Analysis using Welch Method

The Welch method is a power spectrum density estimator that applies the periodgram. It is based on Bartlett's idea of splitting of the data into segments and finding the average of their priodograms. The difference is that the segments are overlapped, usually by 50% or 75%, and the data within the segments are windowed. If *L* is the length of the segments, the *i^th^* segment is denoted by ${\left\{{\text{x}}_{\text{i}}\left[\text{n}\right]\right\}}_{0}^{\text{L}-1},$ and the offset of the successive sequences is *D* samples, then:

(5) N=L+D(k−1)

where *N* is the total number of observed samples and *K* = *N/L*, and if there is 50% overlap, *K* = *2N/L* − *1*.

The *i^th^* sequence is:

(6) $$\begin{array}{cc} x_{i}\left[n\right]=x\left[n+\left(i-1\right)D\right], & n\in \left\{0,1,\cdots ,L-1\right\} \end{array}$$

where i = 1, 2,…, K, and its periodogram is defined by:

(7) $${\hat{P}}_{M}^{i}\left(f\right)=1/L{\left|\sum_{n=1}^{L-1}w\left[n\right]x_{i}\left[n\right]e^{j2\pi fn}\right|}^{2}$$

Here, ${\hat{P}}_{M}^{i}\left(f\right)$ is the modified periodogram of the data because the samples x[n] are weighted by a nonrectangular window w[n]. The Welch spectrum estimate is then given by:

(8) $${\hat{P}}_{B}\left(f\right)=1/K(\sum_{i=1}^{K}{\hat{P}}_{M}^{i}\left(f\right))$$

By permitting the overlap of sequences, more segments can be formed than in the case of Bartlett's method. Also, by using the same number of segments, the overlap allows for longer segments. The increased number of segments reduces the variance of the estimators, and the longer segments improve its resolution. Thus, the Welch method generates a more favorable trade-off of reduction in variance for improvement in resolution than Bartlett's method [28].

## 4. Identification Method

**Figure 6.**
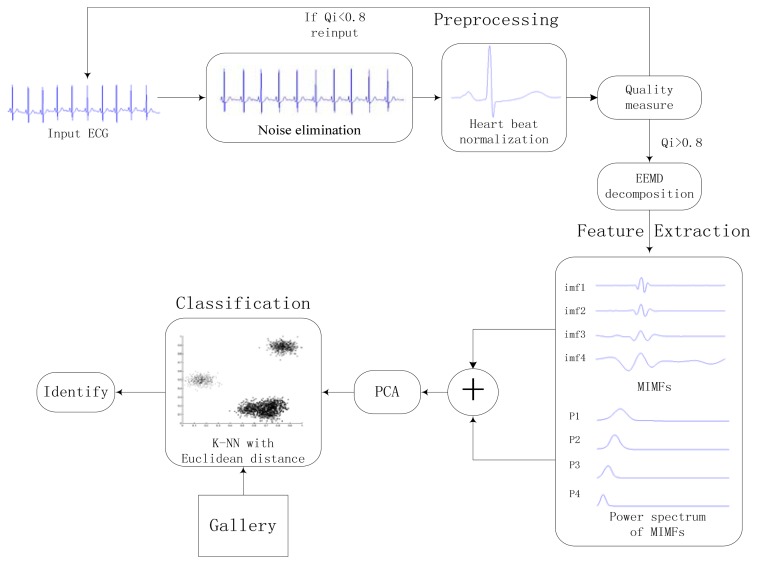
Block diagram of the proposed human identification system.

The proposed approach consists of three stages: preprocessing, feature extraction, and classification. A general block diagram of the proposed identification system is shown in Figure 6. Heartbeat normalization and quality measure is proposed to eliminate the effects of noise and HRV. Then ECG is decomposed into IMFs by EEMD and the power spectrum of each IMF component is estimated by Welch method. The IMFs and power spectrum constitute the feature set, the size of which is reduced substantially by using PCA to reduce the dimensions of the feature coefficients. In end, The K-nearest neighbors (K-NN) method is applied as the classifier.

### 4.1. Preprocessing

**Figure 7.**
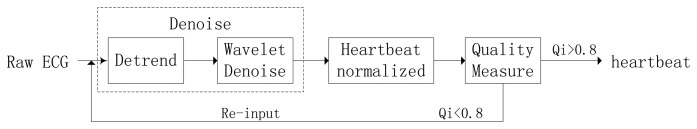
Block diagram of proposed processing scheme.

During the ECG recording, the signal may be corrupted by noise from sources such as baseline drift, power line interference, electrode motion artifacts, and electromyography. This noise alters the waveform of the ECG trace from its original structure. HRV also affects the waveform of the heartbeat signal. To obtain high-quality ECG signals, a robust preprocessing step is proposed to eliminate the effects of noise and HRV. A block diagram of the proposed processing scheme is shown in Figure 7.

#### 4.1.1. Noise Elimination

The noise elimination process consists of two main stages: detrending and wavelet minimax thresholding noise elimination. The detrending method is based on the prior smoothness approach and operates as a time-varying finite impulse response high-pass filter, which can eliminate baseline drift and other low frequency noise. Next, the input ECG signal is decomposed into three levels by biorthogonal spline wavelet, and a threshold is selected on the basis of the minimax thresholding method to remove high frequency noise. The wavelet shrinkage denoising can effectively reduce the noise of non-stationary signal but preserve the local regularity.

The wavelet shrinkage can be decomposed into three steps as follows [29]:

1. Decomposition: compute the wavelet decomposition coefficients of observed signal.
2. Thresholding wavelet coefficients: for each decomposition level (except for the approximation), select a threshold value and threshold function, then apply shrink wavelet coefficients according the threshold value and threshold function.
3. Reconstruction: Wavelet reconstructions based on the modified coefficients, and then restore noiseless signal.

In the wavelet shrinkage, how to select the threshold function and how to select the threshold value are most crucial. Donoho introduced two kinds of threshold functions: “hard threshold function” and “soft threshold function”.

Hard threshold function can be defined as follows:

(9) $$\delta_{\lambda }^{H}\left(x\right)=\left\{\begin{array}{cc} 0 & \left|x\right|\leq \lambda \\ x & \left|x\right|>\lambda \end{array}\right\}$$

Hard threshold function removes the coefficients that are smaller than the threshold and leaves the other ones unchanged.

Soft threshold function can be defined as follows:

(10) $$\delta_{\lambda }^{s}\left(x\right)=\{\begin{array}{ll} 0 & \left|x\right|\leq \lambda \\ x-\lambda & x>\lambda \\ x+\lambda & x>-\lambda \end{array}$$

Soft threshold function also removes the coefficients that are smaller than the threshold but shrink the other ones.

**Figure 8.**
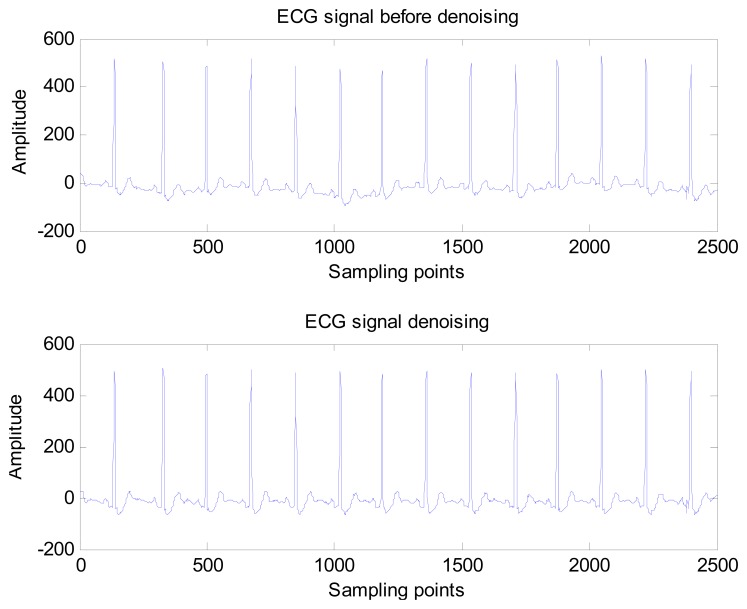
Comparison of ECG signal before and after denoising (ECG record s20051m selected from ST long term).

Donoho and Johnstone proposed a sure shrink thresholding rule based on minimizing unbiased risk estimate. The estimate of the risk can be obtained for a particular threshold value *λ* Minimising the risk gives a selection of the threshold [30]. A comparison of the ECG signal before and after noise elimination is shown in Figure 8.

#### 4.1.2. Heartbeat normalization

**Figure 9.**
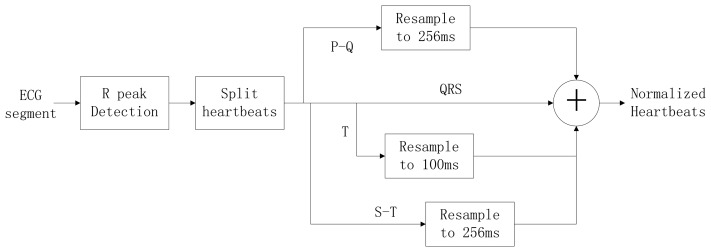
Block diagram of heartbeat normalization process.

Typically, the heart rate of a normal sinus rhythm is 60–100 bpm. During ECG recording, the signal is not constant and can be significantly affected by emotions such as stress and anxiety, or other factors like exercise, shock, or body chemistry, in turn affecting the morphology of the ECG. To eliminate the effects of HRV, a method was proposed to linearly normalize the heartbeat to 75 bpm. A block diagram of the heartbeat normalization process is shown in Figure 9.

To achieve a normalized heartbeat signal, each heartbeat M(i) in an input ECG segment S must be extracted first. QRS complex detection is implemented using the wavelet-based QRS delineation method to determine characteristic point of QRS complexes in ECG signal [31]. Using a biorthogonal spline wavelet to detect the QRS complex of the ECG signal, the signal is decomposed with the equivalent filter of a biorthogonal spline wavelet by Mallat algorithm. The signal singularity's Lipschitz exponent is used to analyse the relationship between the signal singularity (R peak) and the zero-crossing point of the modulus maximum pair of its wavelet transform. After the R-peak detection in the ECG signal, the location of the R peaks are denoted as p(i), i = 1,2,3, …, m. The fiducials of the QRS complex are delineated according to the location of R peak. The characteristic wave intervals of the ECG will be influenced by heart rate changing in some cases [32,33]. The QRS wave shows great stability with the change of the heart rate on the contrary to both the T and P waves which show a variation with the change of the heart rate. According to [34], an adaptive adjusting method is adopted to acquire the characteristic wave intervals of the ECG. Data points are selected from the position of the R peak backward (0.06 × *fs*)th point to forward (0.1 × *fs*)th point, which can be denoted as:

(11) QRS(i)=S[p(i)−0.06×fs:p(i)+0.1×fs]

where *fs* is the ECG recording sample frequency. The waveform of the P-Q duration will change slightly with changes in heart rate, so an adaptive adjusting method for P-Q duration measuring position is selected, which can be calculated using Equation (12):

(12) PQ(i)=S[p(i)−2.24×fs+dt:p(i)−0.06×fs]

where *dt* is a variable threshold depending on the change in heart rate, and it can be expressed as:

(13) $$dt=\{\begin{array}{ll} -10ms & HR<64\text{bpm}\\ +0ms & 65\text{bms}<HR<80\text{bpm}\\ +10ms & 80\text{bms}<HR<95\text{bpm}\\ +20ms & HR>95\text{bpm} \end{array}$$

Experiments have demonstrated an almost linear correlation between the duration of the T wave and the heart rate; thus, the waveform is segmented into two parts after the S wave, which is denoted as s(i) = S[p(i) + 0.1 × fs]. The two segments, called the S-T duration and the T wave, can be obtained using Equations (14) and (15):

(14) ST(i)=S[s(i):s(i)+0.08×RR]

(15) T(i)=S[s(i)+0.08×RR:s(i)+0.4×RR]

**Figure 10.**
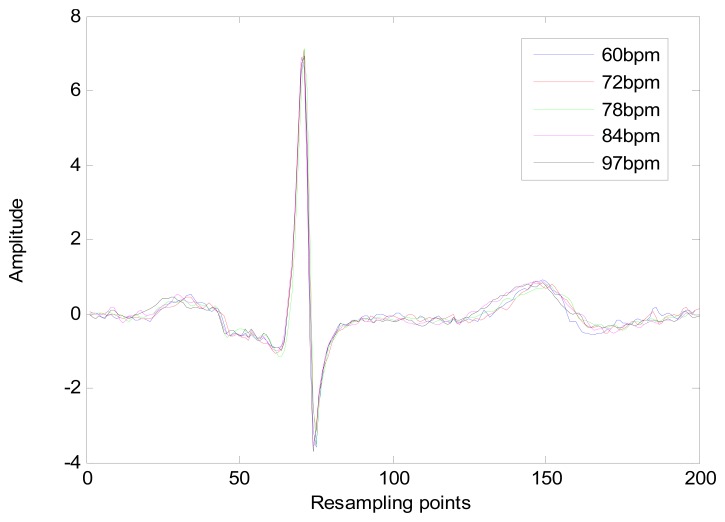
ECG waveform of record 302 m after normalization.

The heart-rate-dependent sections of each heartbeat, P-Q duration, S-T duration, and T wave, are resampled to 216, 100 and 320 ms, respectively, which represent the common lengths of these segments at a resting heart rate. After resampling, these segments are again combined with the heart-rate-independent section, the QRS complex, to recreate the entire heartbeat. The normal length of a resting heartbeat is 800 ms. The amplitude of the heartbeat signal was also normalized to a mean of zero and a standard deviation of one because the amplitude of the ECG signal varies during each ECG recording. The waveform of ECG after normalization is shown in Figure 10. The ECG normalization method effectively eliminates the effects of HRV.

#### 4.1.3. Quality Measure

**Figure 11.**
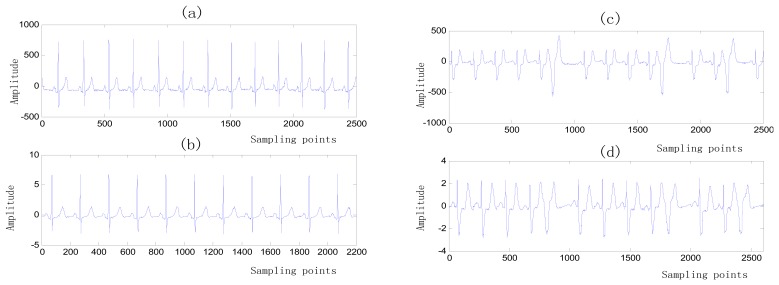
ECG segments from subject in MIT-BIH Arrhythmia database. (**a**)(**b**) High-quality ECG signal with *Q_i_* > 0.8. (**c**)(**d**) Low-quality ECG signal with *Q_i_* < 0.8.

After heartbeat normalization, a quality measure method based on periodicity transforms (PTs) is used to measure the quality of the ECG signal [35]. The M-best algorithm is used to carry out the transform. The heartbeat signal Si extracted from the heartbeat normalization method is combined with a new synthesis signal F, defined as *F*=[*S*_1_,*S*_2_, ⋯,*S_n_*]. During PT, a quality measure Qi is estimated for each synthesis signal and expresses the confidence that the synthesis signal is free of major artifacts. To this end, the PT method is used to project the signals into a sum of periodic sequences. The rationale for the use of PTs is that the synthesis signal is a quasi-periodic signal, and any variation can usually be attributed to the recording error or noise effects. The period of the synthesis signal is 200 sample points; therefore, the PT projects the signal into a periodic sequence period of 200 sample points. The *Q_i_* is then estimated as:

(16) $$Q_{i}=\left\|x_{i}-X_{i}\right\|/\left\|x_{i}\right\|$$

where *x_i_* is the *i*th signal input and *X_i_* is its periodic projection onto the best period approximating the heart rate. *Q_i_* describes the comparative energies between the original and best projected periodic signals. Accordingly, *Q_i_* measures the strength of repetition of the ECG signal. As *Q_i_* for the ECG signal increases, confidence in the collected signal increases. Based on several previous experiments, the threshold was set as 0.8; thus, if *Q_i_* > 0.8, the signal is saved, otherwise another ECG segment is input until the quality of the selected ECG segment can satisfy the threshold requirement [36]. Four ECG segments from subject in MIT-BIH Arrhythmia database with different quality are illustrated in Figure 11.

### 4.2. Feature Extraction Based on EEMD and PSD

EEMD is adaptive and applicable to complex nonlinear and non-stationary series data, such as the ECG signal. When using EEMD, the first decomposed IMF corresponds to the highest frequency, and throughout the sifting process, the frequencies of decomposed IMFs decrease with the residue *R_N_* corresponding to the lowest frequency.

**Figure 12.**
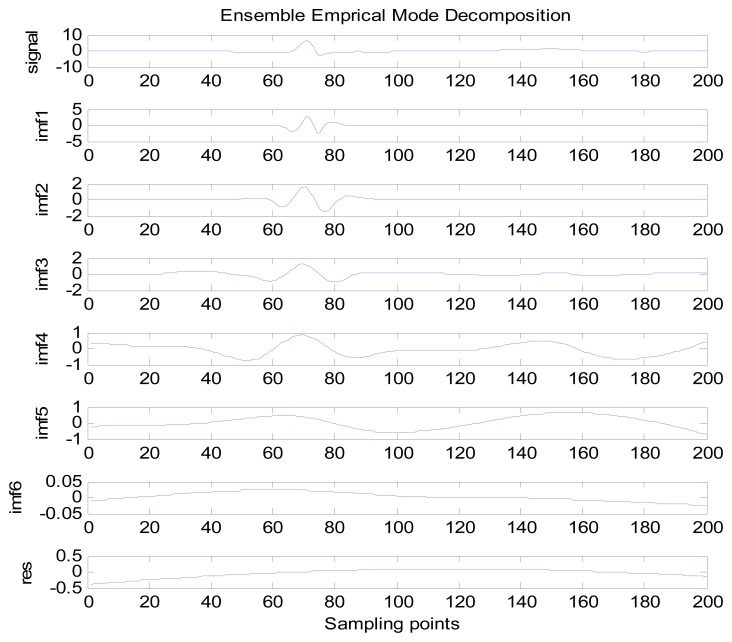
EEMD decomposition result of record 302 m ECG signal.

**Figure 13.**
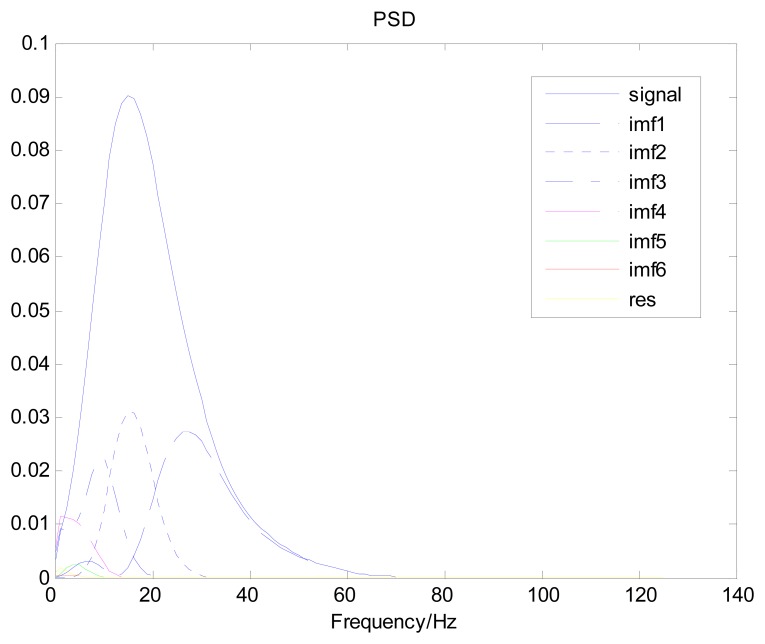
Power spectrum of IMF components for record 302 m.

**Figure 14.**
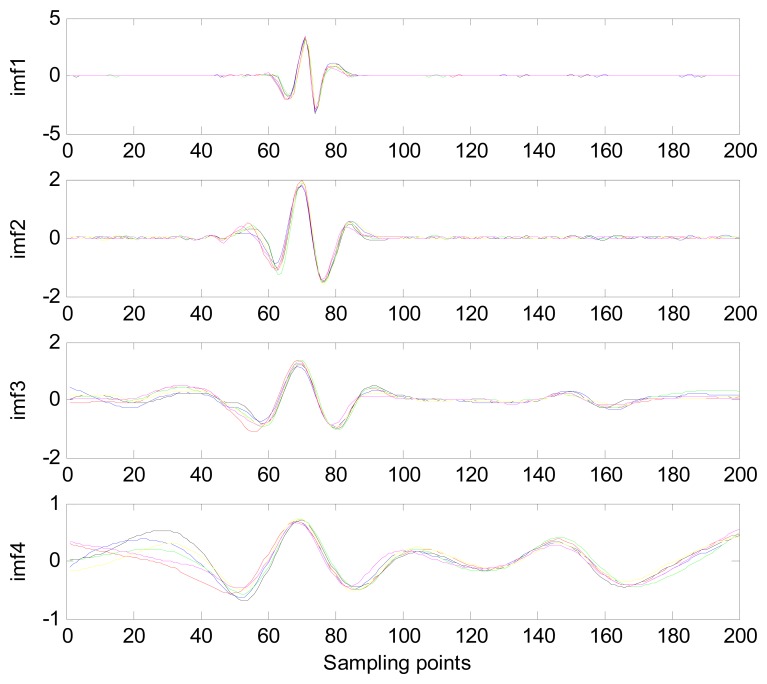
MIMFs from different sessions of record 302 m ECG signal.

**Figure 15.**
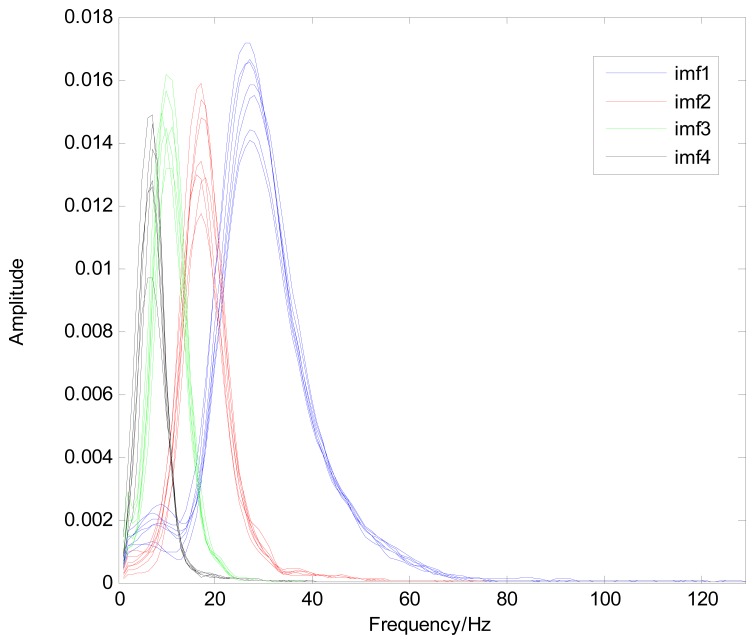
Power spectra of MIMFs from different sessions of record 302 m.

Because the spectral content of the ECG signal is primarily located between 1 and 50 Hz, the main IMFs (MIMFs) can be selected according to the corresponding frequency of each IMF. The spectrum of each IMF was estimated by the Welch spectral analysis method. Figure 12 shows the EEMD result of record 302 m from the MIT-BIH ST change database containing the original signal (signal), IMF components (IMF 1–IMF 6), and the residual component (res). The Welch method was then used to analyze the power spectrum of each IMF component, and the simulation results are shown in Figure 13. The power spectrum of each IMF component reflects the distribution of the signal energy in different frequency scales. The power spectrum shows that most of the signal energy is concentrated in IMFs 1–4. These four IMF components contain the most important information of the heartbeat signal and are considered to be the MIMFs. Consequently, the temporal waveforms and power spectra of these four IMF components were selected as the feature coefficients. The MIMFs and their power spectra for the 302 m ECG record from the MIT-BIH ST change database are shown in Figures 14 and 15, where the heartbeat signals from different sessions have similar shading.

**Figure 16.**
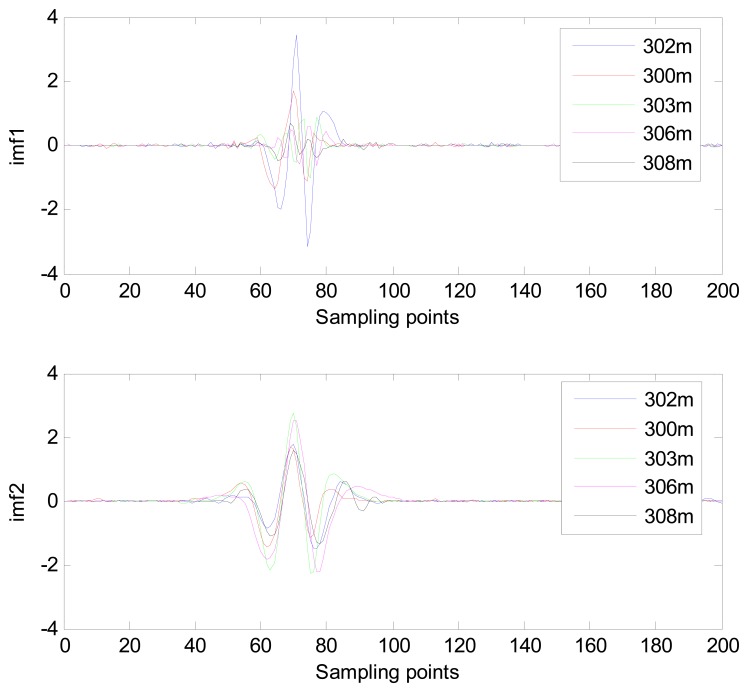
Waveforms of IMF 1 and IMF 2 from different ECG records.

**Figure 17.**
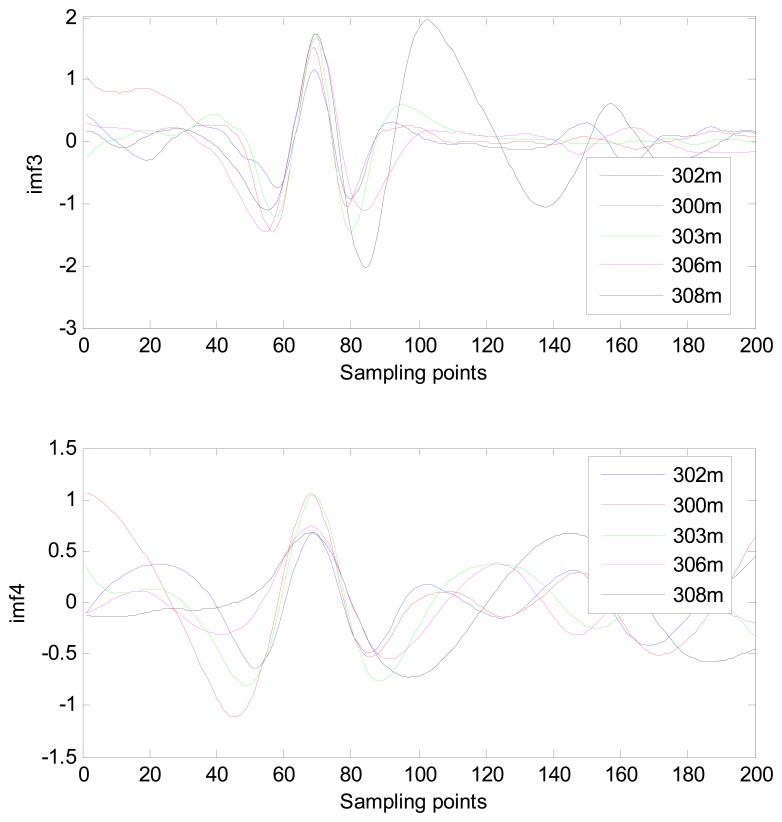
Waveforms of IMF 3 and IMF 4 from different ECG records.

**Figure 18.**
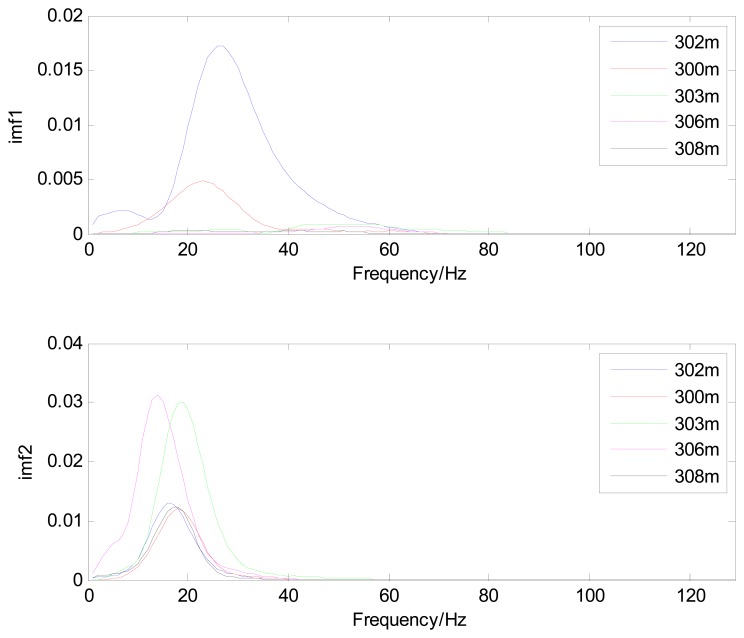
Power spectra of IMF 1 and IMF 2 from different ECG records.

**Figure 19.**
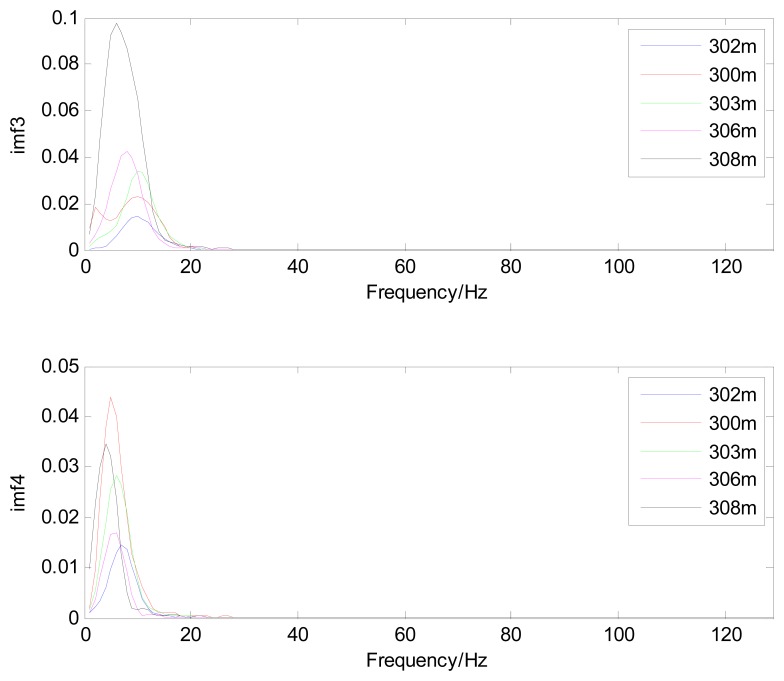
Power spectra of IMF 3 and IMF 4 from different ECG records.

The MIMFs and power spectra of ECG records 300 m, 302 m, 303 m, 306 m, and 308 m from the MIT-BIH ST change database are shown in Figures 16, 17, 18 and 19, where the different ECG records indicate distinctive characteristics. The waveform and the power spectrum of the heartbeat signal are subsequently fused into an eight-dimension matrix as a feature space.

### 4.3. Classification

After feature extraction, the size of the gallery set is reduced substantially by using PCA to reduce the dimensions of the feature coefficients, thus increasing the classification speed. PCA is an unsupervised learning technique that provides an optimal representation of the input data, with respect to least mean square error, in a lower-dimensional space [37]. Given a training set $Z={\left\{Z_{i}\right\}}_{i=1}^{C},$ containing C classes with each class $z_{i}={\left\{z_{ij}\right\}}_{j=1}^{C_{i}}$ consisting of a number of heartbeats *z_ij_*, for a total of $N=\sum_{i=1}^{c}C_{i}$heartbeats, PCA is applied to the training set *Z* to find M eigenvectors of the covariance matrix:

(17) $$S_{\mathrm{cov}}=\frac{1}{N}\sum_{i=1}^{c}\sum_{j=1}^{c_{i}}\left(z_{ij}-\bar{z}\right)\left(z_{ij}-\bar{z}\right)$$

where $\bar{z}=1/N\sum_{i=1}^{C}\sum_{j=1}^{C_{i}}z_{ij}$ is the average of the ensemble. The eigen heartbeats are the first *M(≤N)* eigenvectors corresponding to the highest eigenvalues, denoted as *ψ*. The original heartbeat is transformed into the M-dimension subspace by linear mapping as:

(18) $$y_{ij}=\psi^{T}\left(z_{ij}-\bar{z}\right)$$

where the basis vectors *ψ* are orthonormal. Subsequent classification of heartbeat patterns can be performed in the transformed space. PCA uses an orthogonal transformation to convert a set of variables into a set of principal components, which reduces dimension by retaining those characteristics of the data set that contribute most to its variance. The first four components of IMFs and their power spectrum are constituted the feature coefficients. The IMF feature coefficients matrix is 4 × 200 and their power spectrum coefficients matrix is 4 × 129. PCA is made by eigenvalue decomposition of two coefficients covariance matrix respectively. That is, it is applied twice. PCA reduces dimensions to 4 × 20 respectively. Then 8 × 20 feature matrixes is input into the classifier. The comparison of the application of PCA and without the application of PCA is made. For 10 s ECG input, the application of PCA can reduce the running time above 1 s and improve the efficiency.

The K-NN classifier is a statistics-based tool that is often applied for classification. Its primary objective is to find the k classified features that are most similar to the test features and confirm the categories of the test features according to the k feature categories. The similarity between two features is measured by the Euclidean distance between the two features, where decreasing distance indicates increasing similarity.

## 5. Experimental Results

The experimental procedure used to evaluate the proposed method is shown in Figure 2. The overall identification performance was measured on the basis of ECG segment recognition rates, or the rate of accurate subject identification based on one 10 s ECG segment. Each input 10 s test ECG segment was first subjected to the noise reduction process, followed by heartbeat normalization of the denoised ECG segment to extract the normalized heartbeat signal. The quality measure was then used to measure the quality of the extracted heartbeat signal, and if *Q_i_* of the signal did not satisfy the threshold, another ECG segment was input into the system. If the *Q_i_* of the heartbeat signal did satisfy the threshold, the feature extraction module then extracted the feature coefficients from the signal. This process involved decomposition of the heartbeat signal by the EEMD method. The waveforms and power spectra of IMFs 1–4 were fused to serve as the feature coefficients, forming an eight-dimensional matrix. Because this dimensionality of feature space is considerably high and unsuitable for a cost-efficient system, the PCA is used to reduce the dimensionality. An 8 × 20 matrix is generated using PCA to reduce the dimensionality, and then put into the classifier. Classification is performed by the K-NN classifier using Euclidean distance.

### 5.1. ECG Data

To evaluate the proposed system for subject identification, a series of experiments was conducted using data from three common public databases: the MIT-BIH ST change database, the long-term ST database, and the PTB database [16]. The MIT-BIH ST change database includes 28 ECG recordings of varying lengths, most of which were recorded during exercise stress tests and exhibit transient ST depression. The last five records (323 through 327) are excerpts of long-term ECG recordings and exhibit ST elevation. The sampling frequency of this database is 360 Hz. For this experiment, 15 subject ECG records were selected with measurements representing a range of heart rate for the same subjects. To compare results among the databases, the records were resampled at 250 Hz. Because this database offers only one recording for each subject, several 10 s ECG segments were selected from different sessions in each record with varying heart rates. The gallery set consisted of ECG segments at resting heart rates of 50–70 bpm, and the remaining ECG segments were used to test the performance of the system.

**Figure 20.**
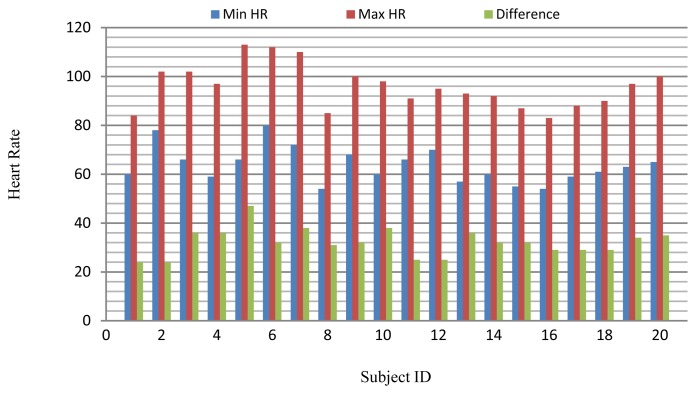
Changes in heart rate for each subject.

The long-term ST database contains 86 lengthy ECG recordings from 80 human subjects, selected to represent a variety of events causing ST segment changes, including ischemic ST episodes, axis-related non-ischemic ST episodes, episodes of slow ST level drift, and episodes containing mixtures of these phenomena. The database was created to support development and evaluation of algorithms for accurate differentiation of ischemic and non-ischemic ST events, as well as basic research into the mechanisms and dynamics of myocardial ischemia. The sampling frequency of this database is 250 Hz. A subset of the database containing 18 subjects with a range of heart rates for the same subject was selected for evaluation. Several 10 s ECG segments from different sessions in each record were chosen to reflect different heart rates. The resting ECG recordings were used to build the gallery set and the remaining ECG segments were used to test system performance. The selected ECG records from the two databases contain a range of heart rates for the same subject as shown in Figure 20.

The PTB database was compiled by the National Metrology Institute of Germany and contains 549 ECG recordings from 294 subjects. This database includes ECG signals for subjects diagnosed with a variety of clinical conditions (e.g., myocarditis, valvular diseases, and myocardial infarction). Recordings that were consistent with the requirements of the proposed simulations were marked as healthy. Every record includes the conventional 12-lead and three-Frank-lead ECG. The sampling frequency of these recordings is 1 kHz; therefore, each recording was resampled at 250 Hz for these experiments. In addition, for every subject in the PTB database, at least two recordings are available that were collected a few years apart. A subset of 12 healthy subjects was formed from the PTB database for these experiments. The criteria for selection of the records were the demonstration of healthy ECG waveforms and at least two recordings. The older recording of each subject was used in the gallery set and the newer one was used to test the system performance.

### 5.2. Experimental Results and Discussion

Each 10 s ECG test segment was subjected to the noise reduction and heartbeat normalization processes, resulting in a normalized heartbeat signal from which the effects of HRV have been eliminated.

#### 5.2.1. Quality Measure

**Figure 21.**
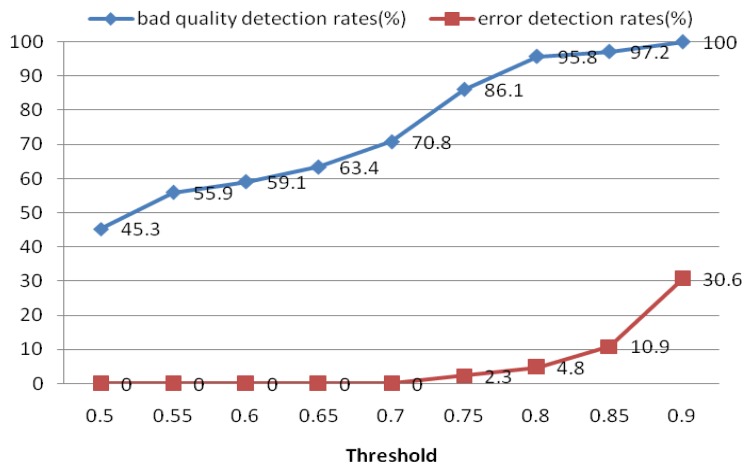
Detection rates for different quality measure thresholds.

The quality measure module was used to measure the quality of the extracted heartbeat signal. For each ECG segment, a quality measure *Q_i_* was estimated using Equation (16). If *Qi* did not satisfy the threshold, another ECG segment was reinput; otherwise, the heartbeat signal was put into the next process. For the quality measure, a threshold of 0.8 was selected to distinguish low- and high-quality ECG records. Figure 21 shows the performance of this process in detecting low-quality records. The results indicate that if the threshold is set too high, it may err and classify some high-quality ECG signals as bad ones. Based on these results, we choose 0.8 as the optimal quality measure threshold. The objective of the quality measure module is to enhance the precision of the system.

#### 5.2.2. Performance under Different EEMD Parameters

Feature coefficients were computed for each heartbeat signal. A number of these parameters could affect the identification rate of the system; therefore it was necessary to validate the selection of these parameters.

**Figure 22.**
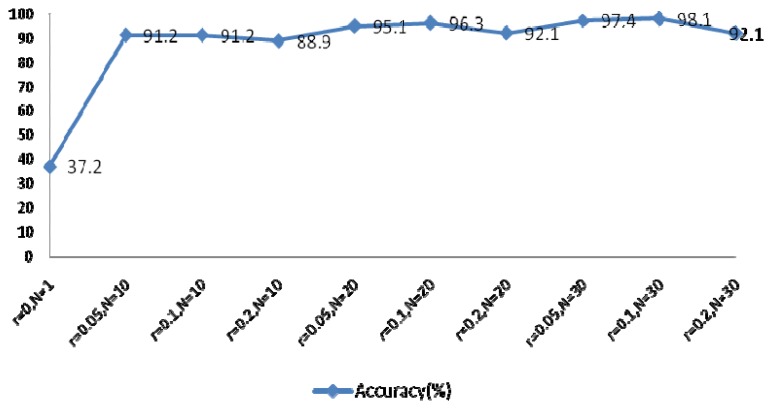
System performance with different *r* and *N* values.

The EEMD method was used to extract the feature coefficients, requiring determination of two parameters: the ensemble number *N* and the ratio of the standard deviation of the white noise to the signal *r*. As *r* increases, the accuracy of the IMF results also increases, however the waveform of the signal will eventually deviate from the normal heartbeat waveform because of the added white noise. The larger *N* becomes, the slower the computation speed. In order to achieve optimal system performance, several groups of parameters were evaluated to determine the optimal *N* and *r*, as shown in Figure 22. This experiment examined the components of IMFs 1–4 using a rectangular window type with a length of 50 sample points, an overlap of 50%, and a fast Fourier transform (FFT) length of 256.

#### 5.2.3. Different Numbers of IMFs

**Figure 23.**
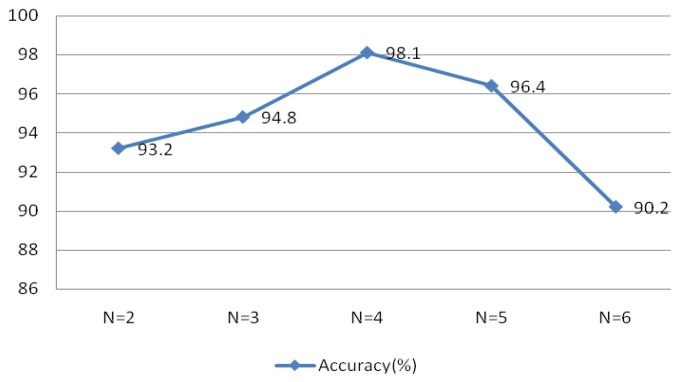
Performance with Different Numbers of IMFs.

Figure 22 indicated optimal system performance when r = 0.1 and N = 30. To verify that the selected MIMFs achieve the best performance, different numbers of IMFs were tested and the resulting system performance is shown in Figure 23. The conditions of this experiment were r = 0.1 and N = 30, the window type was rectangular with a length of 50 sample points, an overlap of 50%, and an FFT length of 256.

Figure 23 validates the theory discussed in Section 4, which indicated that the selected MIMFs should include the most distinctive features to distinguish different subjects.

#### 5.2.4. Window Types

The theory of the Welch spectral analysis method involves dividing the data into segments and finding the average of their periodogram. The type of window, length of window, and the length of FFT are the main factors that may affect the result of the Welch power spectrum. The type of window may affect the estimated power spectrum result; however, the rectangular and Kaiser windows have narrow main lobes and better resolution, but increase the variances of the estimators. Applying the Hamming and Hanning windows, which have wide main lobes and poorer resolution, reduces the variance of the estimators.

**Figure 24.**
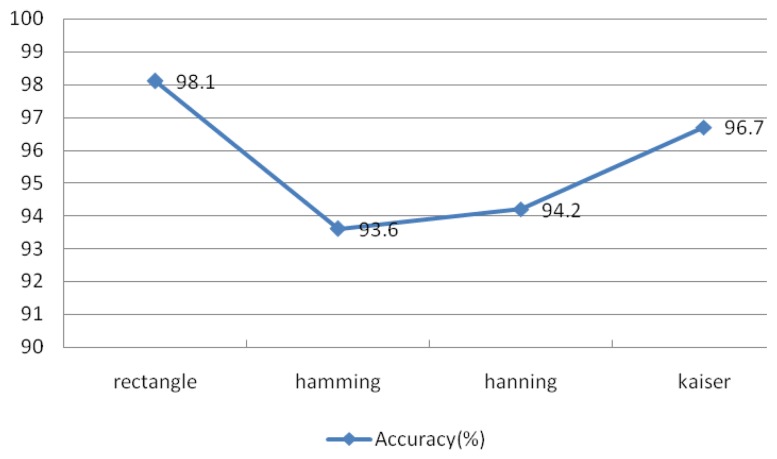
Comparison of different window types.

The observed performance for different types of windows is shown in Figure 24, which suggests that the rectangular and Kaiser windows achieve better performance than the other two types of windows. The rectangular window produced the best identification accuracy at 98.1%. This experiment examined IMFs 1–4 under the conditions *r* = 0.1 and *N* = 30, window length of 50 sample points, an overlap of 50%, and an FFT length of 256.

#### 5.2.5. Window Length

**Figure 25.**
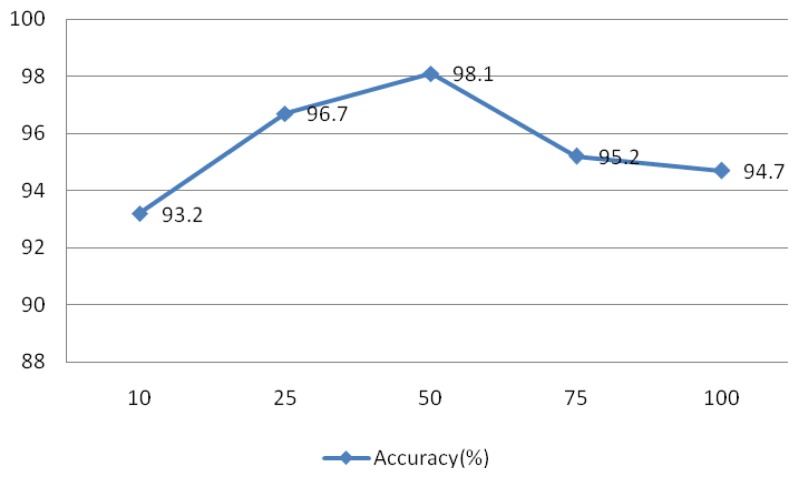
Comparison of different length of window.

The signal was divided into L segments using the Welch method, as L decreases, the variance of the estimators and resolution increases, and vice versa. To test the effect of this parameter on the system, values of L = 10, 25, 50, 75, and 100 were evaluated and the results are shown in Figure 25. This experiment IMFs 1–4 were assessed using r = 0.1 and N = 30, the rectangular window type, an overlap of 50%, and an FFT length of 256.

#### 5.2.6. FFT Lengths

The resolution was dependent on the length of FFT, such increasing length results in increasing resolution. The length of the heartbeat signal is 200 sample points, which is too short compared to other signals.

**Figure 26.**
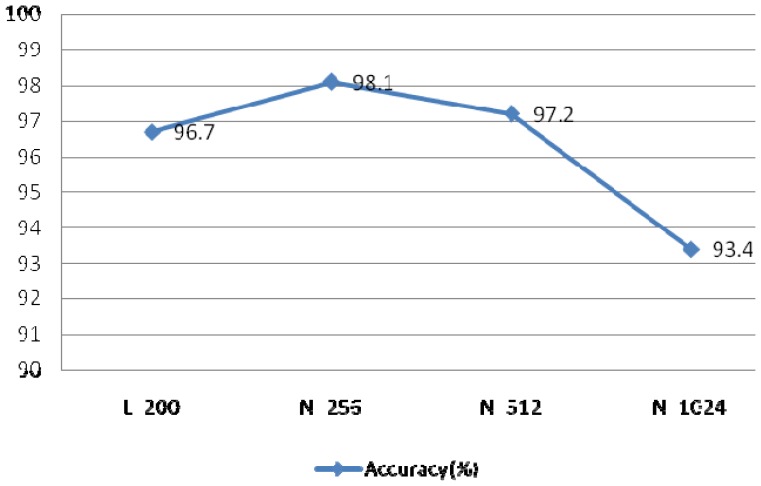
Comparison of different FFT lengths.

If the length of FFT is set to 200, the resolution is poor and the curve is rough. In order to improve the resolution, the length can be set longer than the length of heartbeat signal. A comparison of different FFT lengths of is shown in Figure 26. This experiment evaluated IMFs 1–4 at r = 0.1 and N = 30, using the rectangular window type, an overlap of 50%, and a window length of 50 sample points.

#### 5.2.7. Different Overlaps

**Figure 27.**
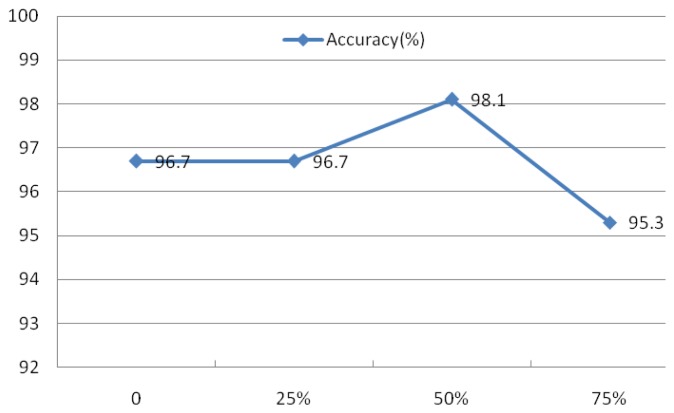
Comparison of different overlaps.

An experiment was also conducted to assess the advantage of the subsequence overlap from the Welch method on system performance. The evaluation of IMFs 1–4 was conducted using *r* = 0.1 and *N* = 30, the rectangular window type, a window length of 50 sample points, and an FFT length of 256. The overlap was set at 0, 25%, 50%, and 75%, as indicated in Figure 27, which shows that the overlap has a minimal effect on the identification rates.

Evaluation of these four factors demonstrated that the spectral resolution is the primary factor contributing to system performance. As a result, the rectangular window with a length of 50, a 50% overlap, and a FFT length of 512 were defined as the parameters for the Welch power spectrum estimation method.

#### 5.2.8. Different Features

Based on these experiments, the parameters of the feature coefficients were defined as *r* = 0.1, *N* = 30, IMF components 1–4, rectangular window type, window length of 50 sample points, and FFT length of 256. To compare the performances of the two types of feature coefficients, the performance of the MIMFs, the power spectra of the MIMFs, and the combined feature coefficients were evaluated and the results are shown in Table 2. Feature coefficients of MIMFs are composed of IMF1–IMF4; feature coefficients of power spectrum of MIMFs is composed of power spectrum of IMF1–IMF4; IMF1–IMF4 and their power spectrum constitute the feature coefficients of MIMFs and power spectrum. These results suggest that the fused feature coefficients produce the best identification rates.

#### 5.2.9. Different k in k-nn

**Figure 28.**
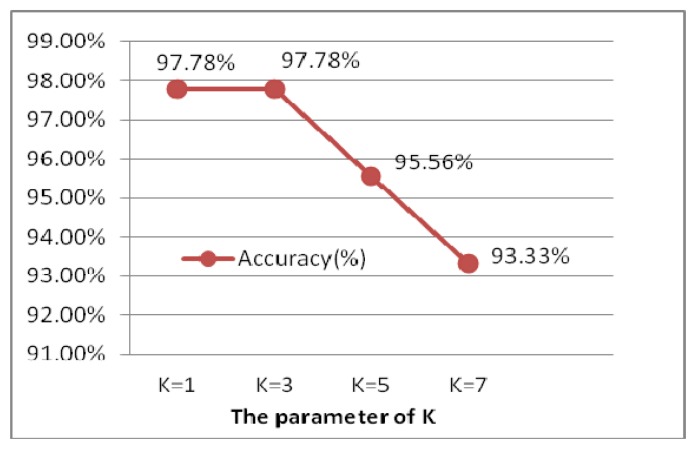
Comparison of different k.

The experiment is performed for the optimal selection of k parameters in k-nn, which is a non-parametric method for classifying objects based on closest training examples in the feature space. The choice of k depends upon the data; generally, k is a positive integer, typically small. The special case (*i.e.*, when k = 1) is called the nearest neighbor algorithm and the object is simply assigned to the class of its nearest neighbor [38]. In order to determine to the optimal k value, k is chosen from 1 to 7 by 2 every step, illustrated as Figure 28. When k is 1 and 3, the ECG identification system achieves the best result of 99.78%. Larger values of k reduce the effect of noise on the classification, but make boundaries between classes less distinct. k = 1 is the optimal selection, which is actually the nearest neighbor algorithm.

#### 5.2.10. Different Time Intervals of ECG

Another experiment has been done to illustrate the influence of the different time intervals. Three different time intervals, 5 s, 10 s and 15 s are chosen.

**Figure 29.**
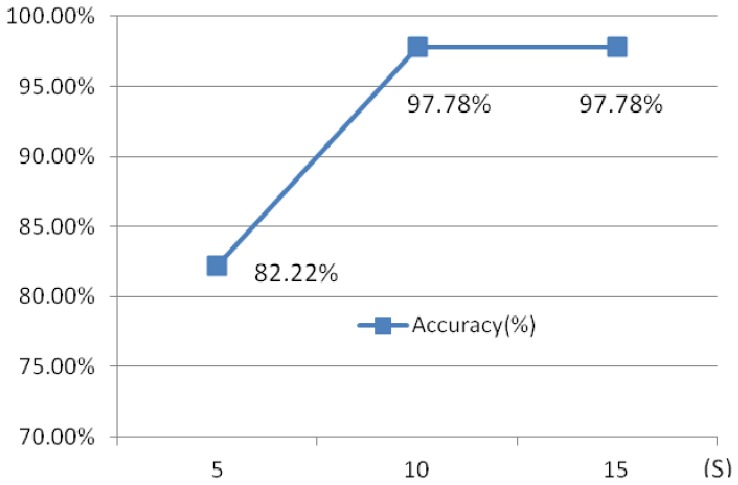
Comparison of different time intervals of ECG.

Figure 29 shows the identification rates which indicate that the 10 s ECG segment has a higher accuracy than 5 s test segment, while 15 s ECG segment and 10 s test ECG segment has a similar accuracy, however, the later requires a longer testing time. 10 s time intervals is optimal selection.

#### 5.2.11. Different Dimension Reduction Methods

**Figure 30.**
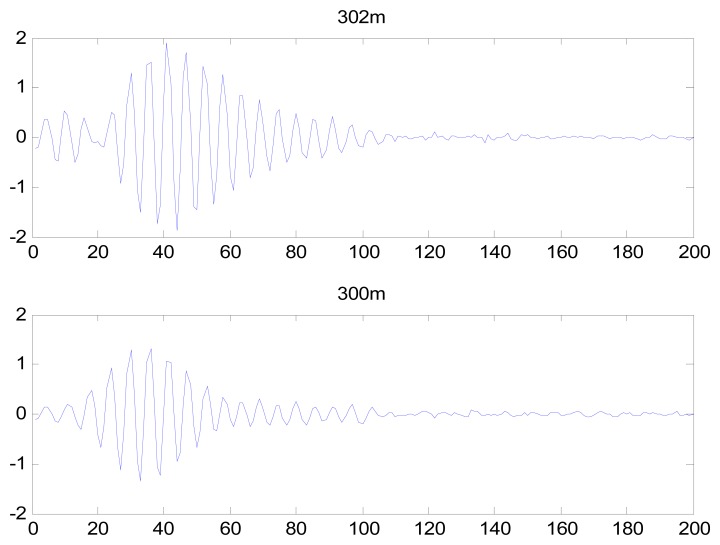
DCT coefficients of IMF 1 from records 300 m and 302 m.

After completion of the feature extraction process, PCA was used to reduce the dimensionality of the heartbeat signal feature coefficients. Using the first 20 coefficients after PCA and converting the eight-dimensional feature matrix into an 8 × 20 matrix, the identification rate was 98.1%. DCT and LDA are two well-known dimensionality reduction tools. LDA is a supervised learning technique that can reduce the dimensionality to C-1, where C is the number of subjects in the gallery. The LDA generated an 8 × 44 matrix, resulting in an identification rate of 76.9%. DCT has an energy compression property, resulting in DCT coefficients of near-zero values; therefore, these values can be dropped to reduce the dimensionality of the feature coefficients. The identification rate using DCT was low. The DCT coefficients of IMF 1 from records 302 m and 300 m are shown in Figure 30, which demonstrate that the DCT did not effectively reduce the dimensionalities of the feature coefficients, and that the DCT coefficients did not exhibit adequately distinct characteristics for individual identification. Thus, PCA was selected to reduce the feature space into an 8 × 20 matrix.

Identification performance with respect to larger database is made. Another 90 subjects selected randomly from the above databases are used to evaluate the performance of biometric systems. The identification results, shown in Table 3, also indicate the identification system achieves better performance and the results are also very encouraging across the larger database.

## 6. Conclusions

ECG is the reflection of electrical changs on the skin that are caused by the eart muscle olarization during each heartbeat, which explain the lectrical ctivity of the eart ver a period of ime and is a body-internal signal. In this paper the use of a proposed EEMD-based ECG feature extraction method for human identification was evaluated. A robust preprocessing scheme was used to compensate for noise, HRV, and low-quality ECG signals. EEMD and Welch spectral analysis were applied to each single-lead ECG signal to extract morphological and spectral information. These two kinds of features were fused and their dimensionality reduced using the PCA method. The system was validated using 45 subjects from three public ECG databases at a variety of heart rates, and the results demonstrate the feasibility of the proposed method as a biometric system. The proposed method performed ideally compared to other methods, achieving a high recognition rate.

ECG identification technology is an emerging new biometric modality. Great progress has been made in the research about ECG biometrics, yet there are several open questions. These include factors associated with the ECG signal collection, heartbeats affected by different cardiac irregularities, *etc.* The databases used in this paper are typical chest ECGs, while lower quality ECGs can be acquired from the hands or at more convenient locations [39]. Chan proposed a scheme that ECG were recorded using a pair of 0.5-in Ag–AgCl button electrodes that are held on the pads of the subject's thumbs using their index fingers [8]. In future, the ECG database should be built containing a large number of people with varying age, collection position, emotional state and different cardiac irregularities. How to extract the features and build classifiers should be further studied to be applicable to more noisy and complex ECGs.

## Figures and Tables

**Table 1.** Previously reported ECG analysis approaches.

**Authors**	**ECG Database**	**Feature Extraction**	**Classification**	**Performance**
Biel *et al.*	Self-collected	Uses a SIEMENS ECG apparatus to record medical diagnostic features	PCA	98%
Shen *et al.*	20 MIT/BIH	Seven fiducial features from P, QRS, and T waves	Template matching DBNN	100%
	168 MIT/BIH	17 fiducial features from P, QRS, and T waves		95.3%
Wang *et al.*	29 MIT/BIH PTB	Extract analytic features and appearance features from ECG fiducial	PCA	100%
			LDA	92.4%
Li Wang *et al.*	Self collected	Use difference threshold method to extract eight duration and amplitude features	BP/RBF	100%
Chiu *et al.*	30(8) QT	Wavelet decomposition to extract feature coefficients	Euclidean distance measure	100%
				81%
Chan *et al.*	30 Self-collected	Heart beat ECG waveform fuse wavelet coefficients	PRD CCORRWDIST	95%
Plataniotis *et al.*	14 MIT/BIH	AC/DCT AC/LDA	Euclidean distance	92.8%
			Gaussian log likelihood	100%

**Table 2.** Performance with different feature coefficients.

**Feature coefficients**	**Performance**
MIMFs	95.3%
Power spectrum of MIMFs	80.9%
MIMFs and Power spectrum	98.1%

**Table 3.** Performance using three public ECG databases.

**Database**	**Number of Subjects (records)**	**Wide Range of HR**	**Accuracy**
MIT-BIH ST change	25	Yes	98.00%
Long-Term ST	40	Yes	95.75%
PTB	25	No	96.00%
Total	90	Yes	95.56%

## References

[1] Jain A.K., Ross A., Prabhakar S. (2004). An introduction to biometric recognition. IEEE Trans. Circ. Syst. Video T..

[2] Biel L., Pettersson O., Philipson L., Wide P. (2001). ECG analysis: A new approach in human identification. IEEE Trans. Instrum. Meas..

[3] Shen T.W. (2005). Biometric Identity Verification Based on Electrocardiogram (ECG). Ph.D. Thesis.

[4] Shen T.W., Tompkins W.J., Hu Y.H. One-Lead ECG for Identity Verification.

[5] Wang Y., Plataniotis K.N., Hatzinakos D. Integrating Analytic and Appearance Attributes for Human Identification from ECG Signals.

[6] Wang L. (2005). The Research Based on the ECG Human Identification Technology. M.S. Thesis.

[7] Chiu C.-C., Chuang C.-M., Hsu C.-Y. A Novel Personal Identity Verification Approach Using a Discrete Wavelet Transform of the ECG Signal.

[8] Chan A.D.C., Hamdy M.M., Badre A., Badee V. (2008). Wavelet distance measure for person identification using electrocardiograms. IEEE Trans. Instrum. Meas..

[9] Plataniotis K.N., Hatzinakos D., Lee J.K.M. ECG Biometric Recognition without Fiducial Detection.

[10] Agrafioti F., Hatzinakos D. ECG Based Recognition Using Second Order Statistics.

[11] Zhao Z., Yang L. ECG Identification Based on Matching Pursuit.

[12] Shen J., Bao S., Yang L., Li Y. (2011). The PLR-DTW Method for ECG Based Biometric Identification.

[13] Matta R., Lau J.K.H., Agrafioti F., Hatzinakos D. Real-Time Continuous Identification System Using ECG Signals.

[14] Sörnmo L., Laguna P. (2005). Bioelectrical Signal Processing in Cardiac and Neurological Applications.

[15] Grauer K. (1998). Practical Guide to ECG Interpretation.

[16] MIT-BIH Database. http://physionet.org/physiobank/database/#ecg.

[17] Huang N.E., Shen Z., Long S.R., Wu M.C., Shih H.H., Zheng Q., Ye N.-C., Tung C.C., Liu H.H. (1998). The empirical mode composition and the Hilbert spectrum for nonlinear and non stationary time series analysis. Proc. Roy. Soc. Lond. A..

[18] Wu Z., Huang N.E. (2004). A study of the characteristics of white noise using the empirical mode decomposition method. Proc. Roy. Soc. Lond. A..

[19] Flandrin P., Rilling G., Goncalves P. (2004). Empirical mode decomposition as a filter bank. IEEE Signal Process. Lett..

[20] Huang N.E., Wu Z. (2009). Ensemble empirical mode decomposition: A noise-assisted data analysis method. Advan. Adapt. Data Anal..

[21] Narsimha B., Suresh E., Punnamchandar K., Reddy M.S. Denoising and QRS Detection of ECG Signals Using Empirical Mode Decomposition.

[22] Bouabida Z., Slimane Z.E.H., Reguig F.B. Detection of QRS Complex in Electrocardiogram Signal by the Empirical Mode Decomposition.

[23] Das M.K., Ari S., Priyadharsini S. On an algorithm for detection of QRS complexes in noisy electrocardiogram signal.

[24] Kouchaki S., Dehghani A., Omranian S., Boostani R. ECG-Based Personal Identification Using Empirical Mode Decomposition and Hilbert transform.

[25] Omar M.O.A., Mohamed A.S.A. Application of the Empirical Mode Decomposition to ECG and HRV Signals for Congestive Heart Failure Classification.

[26] Chang K.-M. Ensemble Empirical Mode Decomposition Based ECG Noise Filtering Method.

[27] Chang K.-M. (2010). Arrhythmia ECG noise reduction by ensemble empirical mode decompostion. Sensors.

[28] Madisetti V. (1999). The Digital Signal Processing Handbook.

[29] Taswell C. (2000). The what, how, and why of wavelet shrinkage denoising. Comput. Sci. Eng..

[30] Donoho D.L., Johnstone I.M. (1995). Adapting to unknown smoothness via wavelet shrinkage. J. Amer. Statist. Assn..

[31] Zheng X., Li Z., Shen L., Ji Z. Detection of QRS Complexes Based on Biorthogonal Spline Wavelet.

[32] Singh Y.N., Gupta P. ECG to Individual Identification.

[33] Tawfik M.M., Selim H., Kamal T. Human Identification Using Time Normalized, QT Signal and the QRS Complex of the ECG.

[34] Ye J., Zheng C., Huang Y. (1999). The characteristic wave intervals of exercise ECG influenced by changing of heart rate (in Chinese). J. Biomed. Eng..

[35] Sethares W.A., Staley T.W. (1999). Periodicity transforms. IEEE Trans. Signal Process.

[36] Agrafioti F., Hatzinakos D. Signal Validation for Cardiac Biometrics.

[37] Abdi H., Williams L.J. (2010). Principal component analysis. WIREs Comp. Stat..

[38] Everitt B.S., Landau S., Leese M., Stahl D. (2011). Miscellaneous Clustering Methods, in Cluster Analysis.

[39] Odinaka I., Lai P.-H., Kaplan A.D., O'Sullivan J.A., Sirevaag E.J., Rohrbaugh J.W. (2012). ECG biometric recognition: A comparative analysis. IEEE Trans. Inform. Forensic Secur..

